# A Method for Rapid Screening of Anilide-Containing AMPK Modulators Based on Computational Docking and Biological Validation

**DOI:** 10.3389/fphar.2018.00710

**Published:** 2018-07-03

**Authors:** Simon W. F. Mok, Wu Zeng, Yuzhen Niu, Paolo Coghi, Yujun Wu, Wai Man Sin, Sio Ian Ng, Flora Gordillo-Martínez, Jia Yin Gao, Betty Y. K. Law, Liang Liu, Xiaojun Yao, Vincent K. W. Wong

**Affiliations:** ^1^State Key Laboratory of Quality Research in Chinese Medicine, Macau University of Science and Technology, Macau, Macau; ^2^State Key Laboratory of Applied Organic Chemistry and Department of Chemistry, Lanzhou University, Lanzhou, China

**Keywords:** AMPK, anilides, virtual screening, cytotoxicity, Lipinski’s rule of five

## Abstract

Adenosine 5′-monophsphate-activated protein kinase (AMPK) is a crucial energy sensor for maintaining cellular homeostasis. Targeting AMPK may provide an alternative approach in treatment of various diseases like cancer, diabetes, and neurodegenerations. Accordingly, novel AMPK activators are frequently identified from natural products in recent years. However, most of such AMPK activators are interacting with AMPK in an indirect manner, which may cause off-target effects. Therefore, the search of novel direct AMPK modulators is inevitable and effective screening methods are needed. In this report, a rapid and straightforward method combining the use of *in silico* and *in vitro* techniques was established for selecting and categorizing huge amount of compounds from chemical library for targeting AMPK modulators. A new class of direct AMPK modulator have been discovered which are anilides or anilide-like compounds. In total 1,360,000 compounds were virtually screened and 17 compounds were selected after biological assays. Lipinski’s rule of five assessment suggested that, 13 out of the 17 compounds are demonstrating optimal bioavailability. Proton acceptors constituting the structure of these compounds and hydrogen bonds with AMPK in the binding site appeared to be the important factors determining the efficacy of these compounds.

## Introduction

Adenosine 5′-monophsphate-activated protein kinase is a key molecular sensor of cellular energy level which maintains homeostasis by responding to the alternation of cellular AMP:ATP ratio. Disruption of such bioenergetic balance is the mechanistic culprit behind a spectrum of disorders (Kuhajda, 2008; Salminen et al., 2011; Day et al., 2017) making AMPK a novel therapeutic target(Gasparrini et al., 2016). For example, metformin, member of the biguanides family sharing the chemical structure HN(C(NH)NH2)2, can interact with LKB1, the upstream kinases of AMPK (Fryer et al., 2002; Shaw et al., 2005) or inhibit complex I of the mitochondrial respiratory chain (Doran and Halestrap, 2000) to reduce blood glucose level which serve as a first-line therapy for type II diabetes (Maruthur et al., 2016). Another category of organic compound demonstrating similar antidiabetic effects is thiazolidinediones which contains thioether- and amine-containing 5-membraned heterocyclic diketone ring as functional group, such as pioglitazone and rosiglitazone, which upregulate AMPK by inhibiting respiratory complex I (Brunmair et al., 2004). Other phytochemicals isolated from natural herbs like ginsenoside Rb1 (Shen et al., 2013), and the polyphenols berberine (Lee et al., 2006) and curcumim (Zheng and Ramirez, 2000), can also reprogram the energy metabolism via the manipulation of AMP level by inhibiting mitochondrial ATP production (Kim et al., 2016). Direct AMPK modulators have been reported and applied to clinical practice as well. AICAR, an AMP analog directly interact with and activate AMPK, is used for cardiac ischemic injury therapy (Corton et al., 1995). In addition, emerging findings are revealing the therapeutic potential of alkaloid-based AMPK activators in the control of tumorigenesis. Natural alkaloid compounds, for example hernandezine, thalidezine, and dauricine, can induce autophagic cell death of drug-resistant cancers through the activation of AMPK activities (Law et al., 2014, 2016, 2017a). Other studies demonstrated that the inhibition of AMPK and autophagy can also potentiate cytotoxicity toward some cancers (Lu et al., 2014; Zhou et al., 2017).

Although AMPK modulators appeared to be a prominent therapeutic strategy for various medical conditions, the accompanying side-effects and other practical issues arouse significant concern. For example, the potential use of metformin in controlling tumorigenesis is constrained by LKB1-deficient cancers (Sanchez-Cespedes et al., 2002). In addition, the glucose metabolic machinery has also been reported as off-target of AICAR associated with the AMP mimetic property of the compound *per se* (Vincent et al., 1991, 1992). Accordingly, methods for rapid selection of compounds which are structurally unrelated to AMP and can directly interact with specific binding site of AMPK are urged. However, novel drug development usually takes a decade or even longer before marketing approval can be granted (Merino et al., 2010). Such demanding process generally consume more than $2 billion USD for investment (DiMasi et al., 2016) which account for most of the failing cases of new drug development (Hernandes et al., 2010). Therefore, efficient screening method with the use of *in silico* platform for searching potential pharmaceutical candidates based on drug-target binding, together with the use of cellular validation tools, will help to enhance pre-clinical trials. In this study, a 4-month screening process for the search of potential AMPK modulators, including the use of computational docking, IC_50_ cytotoxicity test, fluorescence cell imaging, assays for molecular and biochemical analysis, and Lipinski’s rule of five, was reported. We successfully recognized thirteen direct AMPK modulators from over a million of compounds listed in publicly accessible chemical database which are suitable for further *in vivo* evaluation.

## Materials and Methods

### Molecular Docking

The 3D crystal structure of AMPK for molecular docking was retrieved from the Protein Data Bank [PDB ID code: 4CFE (Xiao et al., 2013)]. The binding site was defined based on known ligand (compound 991) in the AMPK-ligand complex which is a benzimidazole derivative and a small molecular activator of AMPK (Lai et al., 2014). The Protein Preparation Wizard was used to remove crystallographic water molecules, add hydrogen atoms, assign partial charges, and minimize the energy by using the OPLS-2005 force field (Kaminski et al., 2001) and the Glide docking program (LLC, 2015) was used in all the docking calculations. The receptor grid box for docking size and centered on the ligand in the active site of AMPK with the box size of 20 Å × 30 Å × 20 Å using the Receptor Grid Generation protocol of Schrödinger (2015). Small molecule in the database Chemdiv was screened. The Ligprep (Schrödinger, 2015) was utilized to the small molecule preprocessing. In the virtual screening process, all the compounds were docked into the binding site of AMPK based on the following three stages: HTVS (20%), standard precision (SP, 20%) and extra precision (XP, 10%). Compounds with XP score less than -7 kcalmol^-1^ were kept for study. Then the selected compounds were calculated the binary fingerprints followed by a hierarchical clustering based on the Tanimoto similarity metric. Finally, compounds with binding mode from visual inspection and with good scores were chosen for biological activities assay.

Detailed interaction between the compound G945-0637 and AMPK was subjected to further docking examination. The 3D structure of G945-0637 and AMPK was obtained from the Chemdiv database and the Protein Data Bank [PDB ID code 4CFE (Xiao et al., 2013)], respectively. The ligand was preprocessed by LigPrep1 using OPLS-2005 force field (Kaminski et al., 2001) to generate the corresponding low energy 3D conformers of G945-0637. The ionized state of G945-0637 was assigned by using Epik at a target pH value of 7.0 ± 2.0 (LLC, 2015) Again, the Protein Preparation Wizard was used to remove crystallographic water molecules, add hydrogen atoms, assign partial charges and protonation states, and minimize the structure using the OPLS-2005 force field (Kaminski et al., 2001). The minimization was terminated when the root-mean-square deviation (RMSD) reached a maximum value of 0.3 Å. G945-0637 was docked into the binding site of the AMPK using the Glide (LLC, 2015) with the standard precision (SP) scoring mode. The docking grid box was defined by centering on the compound 991 in the AMPK. In molecular docking, 500 poses were generated during the initial phase of the docking calculation, out of which best 100 poses were chosen for energy minimization by 1000 steps of conjugate gradient minimizations.

### Compounds for Bioassay Selected From Computational Docking

All of the tested compounds in this study were purchased from the ChemDiv Company.

### Cell Line

HeLa cervical cancer cell line was obtained from the American Type Culture Collection, ATCC (Rockville, MD, United States). HeLa was maintained in the MEM medium (Gibco, United States) supplemented with 10% fetal bovine serum (Gibco, United States), pen-strep-gln (Gibco, United States) at 37°C with 5% CO_2_.

### Cell Viability Assay

IC_50_ cytotoxicity test of compounds were measured using the 3-[4,5-dimethylthiazol-2-yl]-2,5 diphenyl tetrazolium bromide (MTT) (5.0 mg/ml) assay (Gibco, United States). Cells were seeded and incubated in 96-well plates in the MEM medium with 10% FBS (Gibco, United States), and then exposed to various concentrations of the tested compounds dissolved in DMSO (Tianjin Damao Chemical reagent Factory, Tianjin, China) for 72 h. Subsequently, MTT (10 μL) solution was added to each well and incubated at 37°C for 4 h followed by the addition of 100 μL solubilization buffer (10% SDS in 0.01 mol/L HCl) and incubated overnight. The absorbance at 570 nm was determined by microplate reader (Tecan Infinite M200 PRO) in each well on the next day. The percentage of viable cells was calculated using the following formula: Cell viability (%) = Cells number treated/Cells number DMSO control × 100.

### Cellular Fluorescence Imaging

HeLa cells transfected with GFP-LC3 were treated with compounds with concentration according to their corresponding IC_50_ value and then fixed with 4% of paraformaldehyde (Sigma–Aldrich, Darmstadt, Germany). The formation of GFP-LC3 puncta was examined by fluorescence microscopy (Leica DM2500) and quantitated by fluorescent microscopic analysis (Applied Precision DeltaVision Elite, Applied Precision, Inc., United States) following the autophagy guidelines (Law et al., 2017b). In brief, the percentage of cells demonstrating autophagy responses was calculated by dividing the number of cells with increased GFP-LC3 fluorescence puncta (≥10 dots/cell) over the total number of cells. For each experiment, 1000 cells from randomly selected fields were scored.

### AMPK Phosphorylation Assay (Western Blot Analysis)

HeLa cells (45000/well) were seeded on 6-well plate and pre-incubated with different concentrations of the compounds for 24 h at 37°C. Cells were lysed with RIPA lysis buffer with protease and phosphatase inhibitor cocktails. The supernatant containing the cellular proteins was collected and kept in -20°C for later use. Protein concentrations were determined using the Bio-Rad protein assay (Bio-Rad Laboratories, Inc., Hercules, CA, United States). Equal amount of protein samples (Whole cell, Cytoplasm and Nuclear extraction) were prepared and separated by 10% SDS-polyacrylamide gel electrophoresis (PAGE). After electrophoresis, the proteins were transferred to nitrocellulose membrane which was then blocked with 5% dried milk for 1 h. The membrane was then incubated with the primary antibodies recognizing P-AMPK (Thr172) (CST, United States), AMPK (CST, United States), and β-actin (Santa Cruz Biotechnology Inc., United States) overnight at 4°C. The membrane was further incubated with appropriate HRP-conjugated secondary antibodies for 30 min and positive signal was developed using the ECL Western Blotting Luminol Reagents (Santa Cruz Biotechnology Inc., United States).

### AMPK Kinase Activity

AMPK kinase activity was measured by CycLex^®^ AMPK Kinase Assay Kit (MBL, Japan) according to manufacturing instructions. Recombinant active human AMPK (α1 β1 γ1) was incubated with the indicated concentrations of selected compounds or AMP (10 μM) for 20 min at 30°C in a plate pre-coated with the protein mouse IRS-1. AMPK activity was measured using the anti-mouse phospho-Ser-789 antibody, and peroxidase-coupled anti-mouse IgG antibody (30 min at RT). Finally, conversion of the chromogenic substrate tetra-methylbenzidine was quantified by measuring changes in absorbance at 450/550 nm.

### Lipinski’s Rule of Five

The standard parameters, including molecular weight, partition coefficient, hydrogen bond donors, hydrogen bond acceptors, and ligand binding energy were calculated using Advanced Chemistry Development (ACD/Labs) Software V11.02 (Lipinski et al., 2001).

## Results

### Identification of Potential Binding Partners of AMPK

**FIGURE 1:**
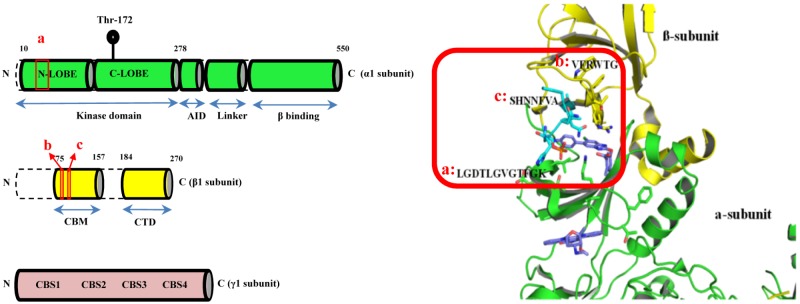
The CBM-KD interface (red box) within AMPK represent the binding site for the computational docking experiment which is a clam-shell-shaped pocket consists of two near-orthogonal orientated β sheets contributed by the CBM of the β1 subunit and the N-lobe of KD of the α1 subunit (CBM, carbohydrate-binding module; KD, Kinase domain).

AMPK is a heterotrimeric complex with the regulatory β- and γ-subunits responsible for sensing cellular energy status (Ferrer et al., 1985; McBride et al., 2009). The KD of AMPK is located within the α-subunit with its enzymatic activity mediated by the phosphorylation of threonine residue 172 (Thr172) (Hawley et al., 1996; Suter et al., 2006). Molecular docking based virtual screening was employed to search for potential AMPK modulators particularly targeting the α1β1γ1 isoform. As shown in **Figure 1** (red box), the binding site was defined as the clam-shell-shaped interface flanked by the β-subunit carbohydrate-binding module (CBM) (a.a. sequence **[b]:** VFRWTG and **[c]:** SHNNFVA) and the N-lobe of the KD of the α1 subunit (a.a. sequence **[a]:** LGDTLGVGTFGK). At first, the inhibitor 991 was extracted from the complex and re-docked into the binding pocket by Glide with SP and XP scoring mode. The RMSDs were 0.41 and 0.68 Å, respectively. Obviously, the Glide docking can reproduce the experimental binding poses. 3000 compounds with good XP score were selected from more than 1,300,000 compounds in the small molecule database Chemdiv via computational docking. Clustering analysis assessing the docking scores and molecular diversity of these compounds further suggested that they are potentially active. Eventually, 250 compounds with binding mode and good scores were recognized by visual inspection. 148 out of 250 of these compounds were commercially available and purchased for biological activities assays.

### Cytotoxicity and the Mechanism of Actions of the Selected Compounds

**FIGURE 2:**
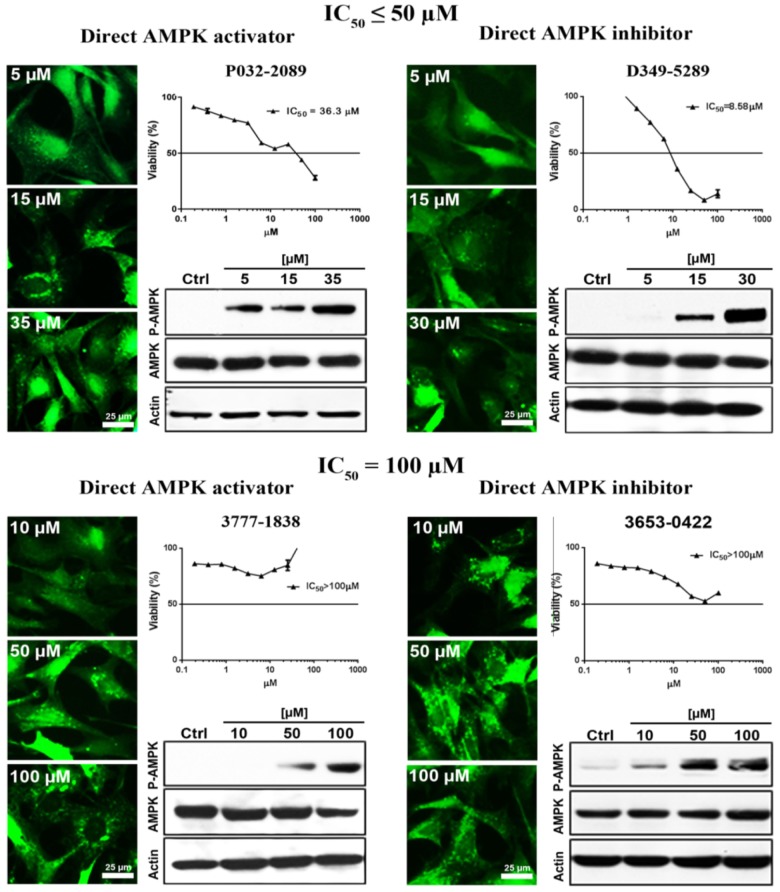
The examined compounds were classified as cytotoxic **(upper)** or non-cytotoxic **(lower)** toward HeLa cells. These compounds either directly activated **(left)** or inhibited **(right)** the AMPK activity. However, all of these compounds induced phosphorylation of AMPK at Thr172 in HeLa cell. In addition, all of the examined compounds induced autophagy activation as demonstrated by the formation of autophagosome (green puncta). Scale bar = 25 μm; Thr172, threonine residue-172 of AMPK.

**FIGURE 3:**
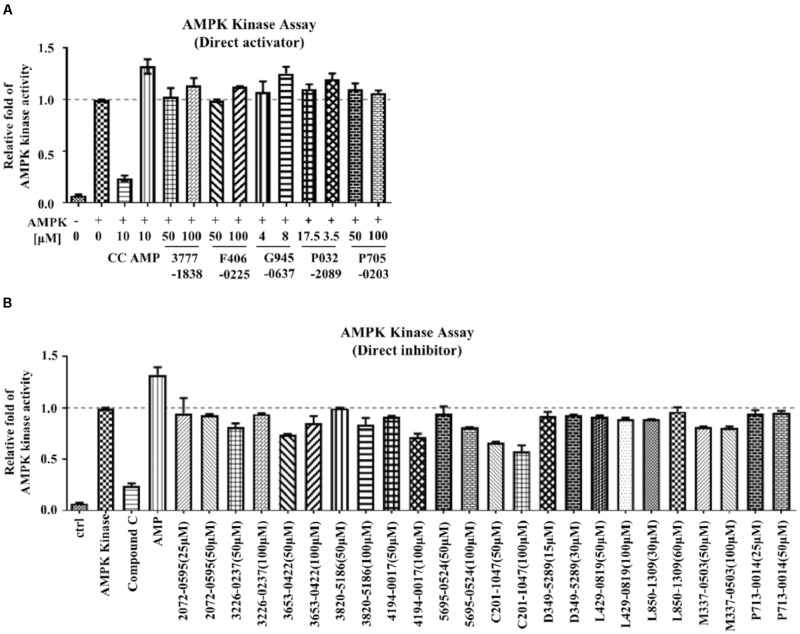
The mechanism of action of the examined compounds assessed by AMPK kinase assay. **(A)** Compounds demonstrated direct activation of AMPK. **(B)** Compounds demonstrated direct inhibition of AMPK.

Conventional MTT assay was then applied to assess the cytotoxicity of the 148 screened compounds with the use of HeLa cancer cells. Compounds demonstrating IC_50_ ≥ 100 μM and ≤50 μM were defined as non-toxic and toxic, respectively. Seventeen compounds were selected after the IC_50_ cytotoxicity test, amongst which 5 of them showed significant cytotoxicity toward our cellular model, while 12 of them were classified as non-toxic (**Figure 2**, **Table 1**, and Supplementary Figure S1). In order to verify the mechanism of action toward AMPK of the compounds selected from the MTT assay, their direct AMPK phosphorylation capacity were analyzed by using AMPK kinase assay kit. We found that all of the 17 compounds could exhibit direct modulatory effect toward AMPK activity. In depth analysis revealed that 2 out of the 5 toxic compounds directly activated AMPK, while the remaining 3 of them inhibited the kinase, via direct interaction (**Figure 3**). For the remaining 12 non-toxic compounds, 3 direct activators and 9 direct inhibitors were verified (**Figure 3**). Since, AMPK is the key regulator of autophagy (Hardie, 2011) the cellular process was used as biological parameter to measure the *in vitro* efficacy of our compounds. Accordingly, fluorescence cell imaging was used to determine the autophagy-inducing effect of these compounds. For the analysis, HeLa cells transfected with the fluorescence autophagy marker GFP-LC3 were treated with the compounds and autophagosome was observed by visual inspection as GFP-LC3 puncta formations. The tested compounds were confirmed as autophagy inducers according to the description in the methodology section. To our surprise, all of the 17 compounds induced significant autophagosome formation and were dependent on the concentration of the added compounds (**Figure 2** and Supplementary Figure S2). Of note, two third of these compounds induced autophagosome formation range from 70 to 90% of the treated cells (**Table 1**) further suggested the significance of their autophagy-inducing effects. In addition, western-blot analysis demonstrated that AMPK phosphorylation at Thr172 of HeLa cells could be induced upon treatment with all of these compounds in a dose-dependent manner (**Figure 2** and Supplementary Figure S3).

**Table 1:** The biological effects induced by the examined compounds.

Compound ID	AMPK Activation	GFP-L3 Puncta Cells (%)	IC_50_ (μM)
**Cytotoxic (IC_50_ < 50 μm)**
G945-0637	Direct activator	80	8
P032-2089	Direct activator	70	3.5
D349-5289	Direct inhibitor	50	8.5
L850-1309	Direct inhibitor	70	50
2072-0595	Direct inhibitor	70	50
**Cytotoxic (IC_50_ = 100 μm)**
F406-0225	Direct activator	70	100
P705-0203	Direct activator	50	100
3777-1838	Direct activator	60	100
C201-1047	Direct inhibitor	60	100
L429-819	Direct inhibitor	60	100
M337-0503	Direct inhibitor	90	100
P713-0014	Direct inhibitor	60	100
3226-0237	Direct inhibitor	50	100
3653-0422	Direct inhibitor	80	100
3820-5186	Direct inhibitor	70	100
4194-0017	Direct inhibitor	70	100
5695-0524	Direct inhibitor	70	100


*The AMPK and autophagy activating ability, as well as the cytotoxicity of the examined compounds toward HeLa cells were studied.*

### Proton Acceptor Is an Important Structural Component in the Selected Compounds Responsible for AMPK Modulation

**FIGURE 4:**
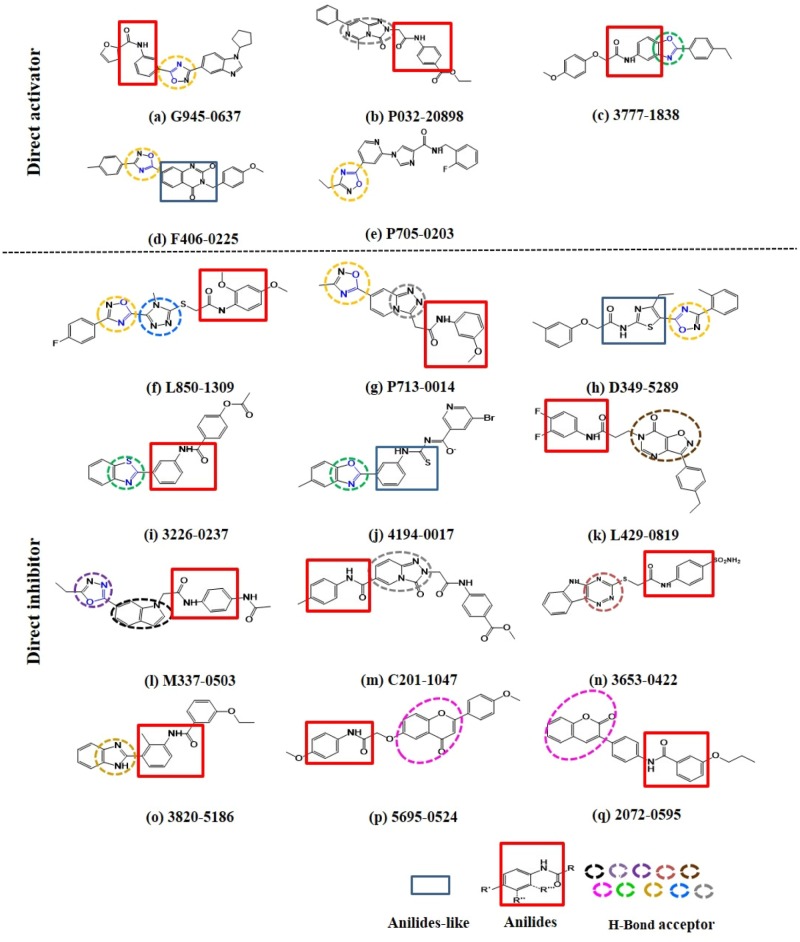
The examined compounds are sharing the common anilides (red box) or anilides-like (blue box) structures. Most of these compounds are either directly or indirectly linked to hydrogen bond acceptor (yellow, green, purple, gray, pink, black, brown, blue, khaki, and orange dashed circle). The chemical structures of more than half of these proton acceptors were sharing similarity by having -N = C-O- (blue colored) in their pentacyclic core.

**FIGURE 5:**
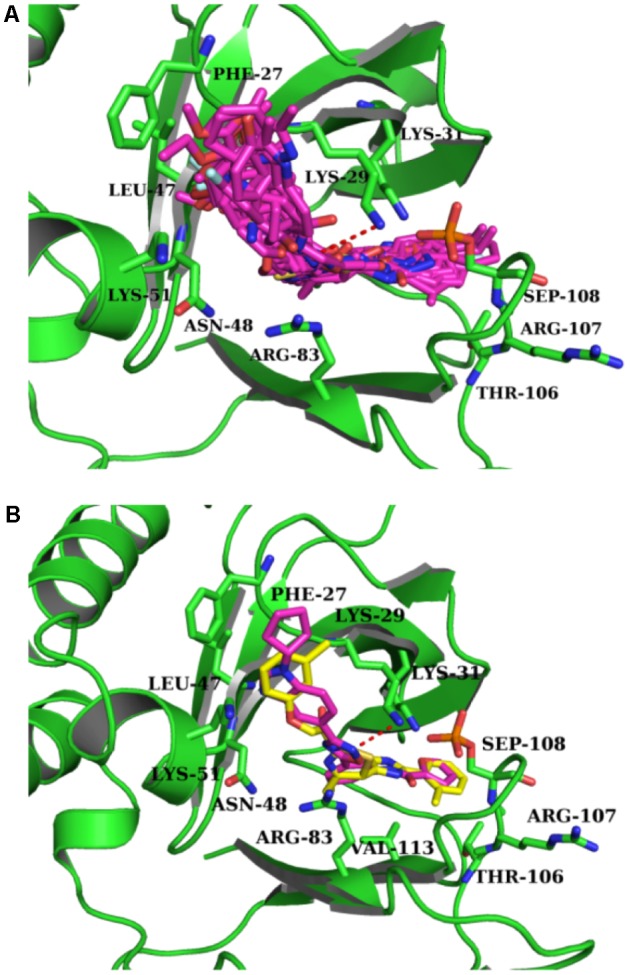
The binding mode of the compounds with AMPK. **(A)** The binding mode of all compounds with AMPK. **(B)** The binding mode of G945-0637 and D349-5289 with AMPK. G945-0637 is labeled by purple and D349-5289 is labeled by yellow; AMPK (green structure) and the key residues around the binding pocket are shown as sticks. The hydrogen bond is labeled as red dashed line.

Intriguingly, 16 out of 17 of the identified compounds **(except e)** are structurally resemblance to each other by sharing the common anilides or anilides-like structures (red and blue boxes) as shown in **Figure 4**. These compounds are anilides or acet- or benz-anilide (anilide analogs) substituted directly or via heteroatom (nitrogen, oxygen, and sulfur) to heterocyclic or aromatic ring. Most of these anilides and the corresponding analogs are directly or indirectly linked to at least one of the following structures: 1,2,4-oxadiazole (yellow circle), 1,3-oxazole/1,3-thiazole (green circle), 1,3,4-oxadiazole (purple circle), 3-oxo-1,2,4-triazol-pyridine and pyrimidine/1,2,4-triazol-pyridine (gray circle), 1,2 and 1,4-benzopyrone (pink circle), 1H-indole (black circle), 7-oxo-isoxazolo-pyrimidine (brown circle), 1,2,4-triazole (blue circle), 1,3-diazole (Khaki circle), and 1,2,4-triazine (orange circle) which can act as scaffold for H-bond formation by acting as proton acceptor due to the presence of highly electronegative atom (i.e., nitrogen and oxygen). The pentacyclic core of proton acceptors 1,2,4-oxadiazole, 1,3-oxazole/1,3-thiazole, and 1,3,4-oxadiazole (yellow, green, and purple circles), resemble each other by sharing the -N=C-O- in the structure. In order to study the interaction between the AMPK protein with its modulators, we docked all the active compounds in the binding pocket of AMPK. Our docking results showed that all compounds shared similar binding poses within AMPK (**Figure 5A**). However, the docking scores of all the compounds (**Table 2**) appeared to have no direct correlation to their activities. On the other hand, all the tested compounds could interact with the Phe27, Lys29, Lys31, Leu47, Lys51, Thr106, Arg107, Ser108, and Val113 side chains of AMPK via its phenyl and cyclopentyl rings. Electrostatic interactions were observed at Lys29, Lys31, Asn48, Arg83, and Ser108 near the α1 subunit of AMPK. We further compared the binding mode of ligand G945-0637 (the relatively active compound) and D349-5289 (compound with no significant activity) to AMPK (**Figure 5B**). The docking scores of these compounds illustrated no significant difference (-7.40 kcal/mol for G945-0637 and -7.59 kcal/mol for D349-5289, respectively). Intriguingly, the binding mode of the two compounds shared similarity while minor difference was found, in which, G945-0637 could form two additional hydrogen bonds with the backbone of Lys29 of AMPK (red dashed line) (**Figure 5B**).

**Table 2:** Structures and physicochemical parameters of the compounds.

	Formula	MW	Log P	H don	H acc	Alert	LBE (Kcal/mol)
	Lipinsky’s rule	<500	<5	<5	<10	0	
(a)	C_25_H_21_N_6_O_3_	439.4	2.89	1	8	0	-7.40
(b)	C_23_H_21_N_5_O_4_	434.4	2.95	1	9	0	-7.52
(c)	C_24_H_22_N_2_O_4_	402.3	4.76	1	6	0	-7.89
(d)	C_25_H_20_N_4_O_4_	440.4	4.66	1	8	0	-8.51
(e)	C_20_H_17_FN_6_O_2_	392.3	2.65	1	7	0	-9.28
(f)	C_21_H_19_FN_6_OS	470.4	5.6	1	10	1	-8.26
(g)	C_18_H_16_N_6_O_3_	364.3	0.98	1	9	0	-7.51
(h)	C_23_H_22_N_4_O_3_S	434.5	5.6	1	7	1	-7.59
(i)	C_22_H_16_N_2_O_3_S	388.4	3.98	1	5	0	-8.98
(j)	C_21_H_15_BrN_4_O_2_S	467.3	4.07	2	6	0	-7.42
(k)	C_22_H_18_F_2_N_4_O_3_	424.4	6.16	1	7	1	-7.98
(l)	C_22_H_21_N_5_O_3_	403.4	3.40	2	8	0	-8.00
(m)	C_24_H_21_N_5_O_5_	459.4	4.07	2	10	1	-8.31
(n)	C_17_H_14_N_6_O_3_S_2_	414.4	0.77	4	9	0	-7.94
(o)	C_23_H_21_N_3_O_2_	371.4	3.6	2	5	0	-7.43
(p)	C_25_H_21_NO_6_	431.4	3.92	1	7	0	-7.76
(q)	C_25_H_21_NO_4_	399.4	5.023	1	5	1	-7.42


*Lipinski’s rule of five analysis: MW (Da), molecular weight; log P, partition coefficient; oxH-don, hydrogen bond donors; oxH-acc, hydrogen bond acceptors; Alert, computational alert according to the rule of five. 0 = no problem detected,1 = poor absorption or permeation are more likely; LBE, ligand binding energy.*

### The Selected Compounds Meet the Criteria of the Lipinski’s Rule of Five

Finally, we have also examined the physicochemical properties of the 17 compounds by applying the Lipinski’s rule of five (Lipinski et al., 2001) in order to evaluate their drug-likeness. Thirteen out of 17 of the compounds (a to e, g, i, j, and l to p) are having their molecular weight and log *P*-value smaller than 500 Da and 5, respectively (**Table 2**). Also, 12 out of 13 of these compounds (except m) are possessing less than 5 H-bond donors and 10 H-bond acceptors (**Table 2**), therefore, are favorable to the development of orally active drugs.

## Discussion

Many of the commonly available pharmaceutical compounds are mechanistically targeting a board range of cellular components or mediators of signaling pathways, including membrane receptors and enzymes (Golebiowski et al., 2003; Hugo Kubiniy, 2008). As such, information directed against intermolecular interactions in the drug-target complex will increase the successful rates of discovering novel therapeutic compounds for the later biological assays (Paolini et al., 2006; Keiser et al., 2007; Whitty, 2008). Here we are interested in compounds or ligands targeting AMPK, as the protein kinase are involved in a variety of diseases. In particular, increasing findings are evidencing the critical role of modulating AMPK activities by natural small molecules in tumorigenesis and neurodegenerations. Also, isoforms of α, β, and γ subunits are existed (i.e., α1, α2, β1, β2, γ1, γ2, and γ3), and the different combinational arrangement of these subunit variants contributed to the formation of heterotrimeric complex isoforms of AMPK. The various isoforms of AMPK exhibit differential functions and are expressed in a cell- or tissue-dependent manner (Birk and Wojtaszewski, 2006), the exploitation of isoform-specific AMPK modulators would encourage the development of more efficient pharmaceutical interventions (Cool et al., 2006; Hawley et al., 2012; Xiao et al., 2013; Zadra et al., 2014). In this study, α1β1γ1 was chosen as the target since it is the most common isoforms expressed in a board spectrum of cell types (Stapleton et al., 1996; Thornton et al., 1998; Cheung et al., 2000). The computational-based virtual screening method used in the current study is an effective way for the search of active compounds from small molecule databases. Such method allows large-scale screening for 10s of 1000s of candidate compounds, but simultaneously maintains specificity of the results by limiting the search in the database for accessible compounds (Shoichet, 2004). In our case, close to 60% (148 out of 250) of the compounds was commercially available avoiding the costly syntheses process.

17 out of the 148 compounds were verified as either toxic or non-toxic. In actual practice, there is no absolute standard for defining the IC_50_ value of a compound to be considered as toxic. Generally, the lower the value, the more cytotoxic is the compound. Here we defined IC_50_ ≤ 50 μM as the cut-off for selecting compounds which are toxic toward the HeLa cancer cells, since generic cancer drugs like paclitaxel, oxaliplatin, docetaxel, and anastrozole, are having their IC_50_ values mostly within the range of 0 μM < IC_50_ < 50 μM (Florento et al., 2012). Therefore, the setting of IC_50_ values for this study provided a reasonable first screening platform after computational docking by avoiding the filtering of an excessive amount of compounds out from the list and at the same time kept an acceptable stringency for the assay. All of the compounds selected by the IC_50_ test could phosphorylate HeLa-isolated AMPK at Thr172 suggested that all of the 17 compounds can directly activate AMPK in living cells. Intriguingly, the AMPK kinase assays controversially demonstrated that, more than half of these compounds were direct AMPK inhibitors. This can be explained by the fact that these direct AMPK inhibitors could target other molecular components in the cellular environment such as the upstream kinases of AMPK with a higher affinity. Therefore, direct AMPK inhibitor may also be able to activate AMPK by targeting other cellular off-targets. In addition, the autophagy-inducing property of these compounds was assessed with cellular fluorescence imaging. HeLa cells were particularly used as the experimental model, since they are flattened in shape and demonstrate discrete cellular compartments which provide suitable platform for observing autophagosome formation. Unexpectedly, all of these compounds, including those direct AMPK inhibitors, could trigger the induction of autophagy. The induction of autophagy by the compounds illustrating direct inhibitory effects could most properly be mediated by molecular pathways, such as the PI3K/AKT/mTOR signaling, bypassing AMPK. In fact, the response of other direct AMPK modulators under cellular environment also demonstrated such complexity. AICAR, an direct AMPK activator and AMP analog, may activate AMP-dependent enzymes, such as fructose-1,6-bisphosphatase (Vincent et al., 1991; Matifat et al., 1997). However, another AMP mimetic Compound-2 (C-2) and its pro-drug C-13 can allosterically activate AMPK without affecting any enzymes regulating AMP (Gomez-Galeno et al., 2010). In addition, the direct AMPK activator PT-1 that binds directly to a specific site in the AMPK isoform α1β1γ1 as demonstrated in computational docking to induce ACC phosphorylation has also been shown to activate AMPK in an indirect manner by inhibiting the mitochondrial function (Jensen et al., 2015). The discrepancies as illustrated in the biological assays emphasized the necessity of using both AMPK kinase assay and cell-based AMPK phosphorylation assessments for validating newly discovered AMPK modulators, which are important procedures in the proposed method. This is further supported by the observation that there is no direct correlation between the binding energy and the activity of the tested compounds. The proposed constituting biological assays are useful for providing experimental data to compensate the crude *in silico* prediction for the activity of the tested compounds acquired from the first docking step.

It is worth noting that, most of the selected compounds (16/17; i.e., around 94%) are anilides or the structural analogs of anilide which are different from the previously reported direct AMPK modulators, such as the small molecules hernandezine and thalidezine in the family of isoquinoline alkaloid, which has been recently discovered by our laboratory (Law et al., 2016, 2017a) or compounds sharing the 4-(2-hydroxypheny)phenyl-side chain and a negatively ionizable group in their structures, for example, the derivatives of alkene oxindole, cyclic benzimidazole, pyrimidine, thienopyridone, and ring-fused imidazole (Giordanetto and Karis, 2012). The molecular function of some patented anilides and their derivatives or analogs are associated with the inhibition of kinase in the Rho and Src families (Feng et al., 2009; Huisman et al., 2016), which points toward its therapeutic role in various disorders including cancers (Itoh et al., 1999). To our knowledge this is the first example demonstrating the capability of anilide-based compounds of functioning as direct AMPK modulators which may suggest new mechanism of action for explaining the pharmacological effects of these compounds. The chemical structures of the identified compounds were subjected to in-depth analysis. Most of them were found to have their anilides or anilide-like nucleus linked to the proton acceptors. 1,2,4-oxadiazole (yellow circle) and oxazole/thiazole (green circle) were the most frequently observed proton acceptors constituting these compounds implying that the chemical structures of these proton acceptors may be related to the functional property of the identified compounds. This notion was supported by the fact that, more than half (c.a. 60%) of the chemical structures of these proton acceptors were sharing similarity by having -N=C-O- in their pentacyclic core (yellow, green and purple circles). The result acquired from the detailed docking analysis of compound **(a)** are in line with these findings as well. The virtual screening suggested that the proton acceptor structures of **(a)** can potentially interact with AMPK at the binding pocket mediated by H-bond formation, which, together with other hydrophobic, polar, and electrostatic interactions between other side chains of **(a)** and AMPK, may most probably contribute to the proper positioning of **(a)** within the binding pocket. Since, **(a)** illustrated the most significant AMPK modulation effects amongst the 17 compounds, which suggested the critical role of proton acceptors within the structures of anilide/anilide-like AMPK modulators. Such observation may provide insight to the development of AMPK modulators with desired pharmacological performance through suitable structural modification of compounds in the anilide group.

It is important to have a brief idea of the oral bioavailability of a newly found compound for referencing the likelihood of further large scale and intensive *in vivo* assessments. Therefore, the drug-likeness of the 17 compounds were analyzed according to the Lipinski’s rule of five which predict the permeability and absorption of a compound and is especially useful for rationalizing compound design via computer-aided drug discovery (Sliwoski et al., 2014). Compounds which do not violate the Lipinski’s rule of five are potentially possessing optimized folding, polarity, and molecular size, therefore, drug-like molecules exhibiting desirable pharmaceutical properties (Zhang and Wilkinson, 2007). Since, the Lipinski’s rule of five is a valid guide for predicting the potentiality of oral exposure for optimized chemical compounds, its purpose in the proposed method served merely to provide preliminary information describing the potential physical properties of the compounds, instead of functioned as a rigid screening criteria. In the current study, we have figured out from the 17 compounds, 12 of them were having the potential to reach the systemic circulation via oral administration. These findings was acquired in 4 months commenced with the virtual screening of over a million of compounds from the database which highlighted the efficacy and practicality of the proposed method.

## Conclusion

We have proposed an efficient method, including the use of computational docking and straightforward biological methods, for the search of AMPK modulators from chemical database. Compounds sharing the anilides/anilides-like structure have been discovered as a new class of AMPK modulator. In addition, positioning of H-bond within these chemical structures determined the mechanism of actions of such compounds providing insight to drug synthesis via structural modifications.

## Author Contributions

LL, XY, VW, and SM conceived and designed the research. SM drafted the manuscript. WZ, YW, YN, PC, WS, and SN performed the experiments. SM, WZ, and PC analyzed the data. WZ, PC, FG-M, JG, and BL edited the article. LL, XY, and VW approved the final version of the article.

## Conflict of Interest Statement

The authors declare that the research was conducted in the absence of any commercial or financial relationships that could be construed as a potential conflict of interest.

## Abbreviations

ACC: Acetyl-CoA carboxylase
AICAR: nucleoside 5-aminoimidazole-4-carboxamide riboside
AMPK: adenosine 5′-monophsphate-activated protein kinase
BBB: blood-brain barrier
C-2/C-13: compound -2/compound-13
CBM: carbohydrate-binding domain
GFP-LC3: green fluorescent protein-light chain 3
H-bond: hydrogen bonds
IRS-1: insulin receptor substrate-1
KD: kinase domain
LKB1: liver kinase B1
PI3K/AKT/mTOR: phosphoinositide-3 kinase/protein kinase-B/mammalian target of rapamycin
PSA: topological polar surface area
