# Supplementary material for: A Method for Rapid Screening of Anilide-Containing AMPK Modulators Based on Computational Docking and Biological Validation
Source: Front Pharmacol. 2018 Jul 3;9:710. doi: 10.3389/fphar.2018.00710 (PMC6037836; doi:10.3389/fphar.2018.00710)
Supplement: Supplementary file 1 [file Image_1.PDF]

Supplementary Material

CIFAL: A Method for Rapid Screening of Anilide-containing AMPK Modulators Based on Computational Docking and Biological Validation

Simon Wing Fai Mok[1], Wu Zeng[1], Yuzhen Niu[2], Paolo Coghi[1], Yujun Wu[1], Wai Man Sin[1], Sin Ian Ng[1], Flora Gordillo-Martínez[1], Jia Ying Gao[1], Betty Yuen Kwan Law[1], Liang Liu[1*], Xiaojun Yao[1*], Vincent Kam Wai Wong[1*]

*Correspondence: Dr. Vincent Kam Wai Wong (bowaiwong@gmail.com), Prof. Xiaojun Yao (xjyao@must.edu.mo), and Prof. Liang Liu (lliu@must.edu.mo), State Key Laboratory of Quality Research in Chinese Medicine, Macau University of Science and Technology.

Supplementary Figure 1

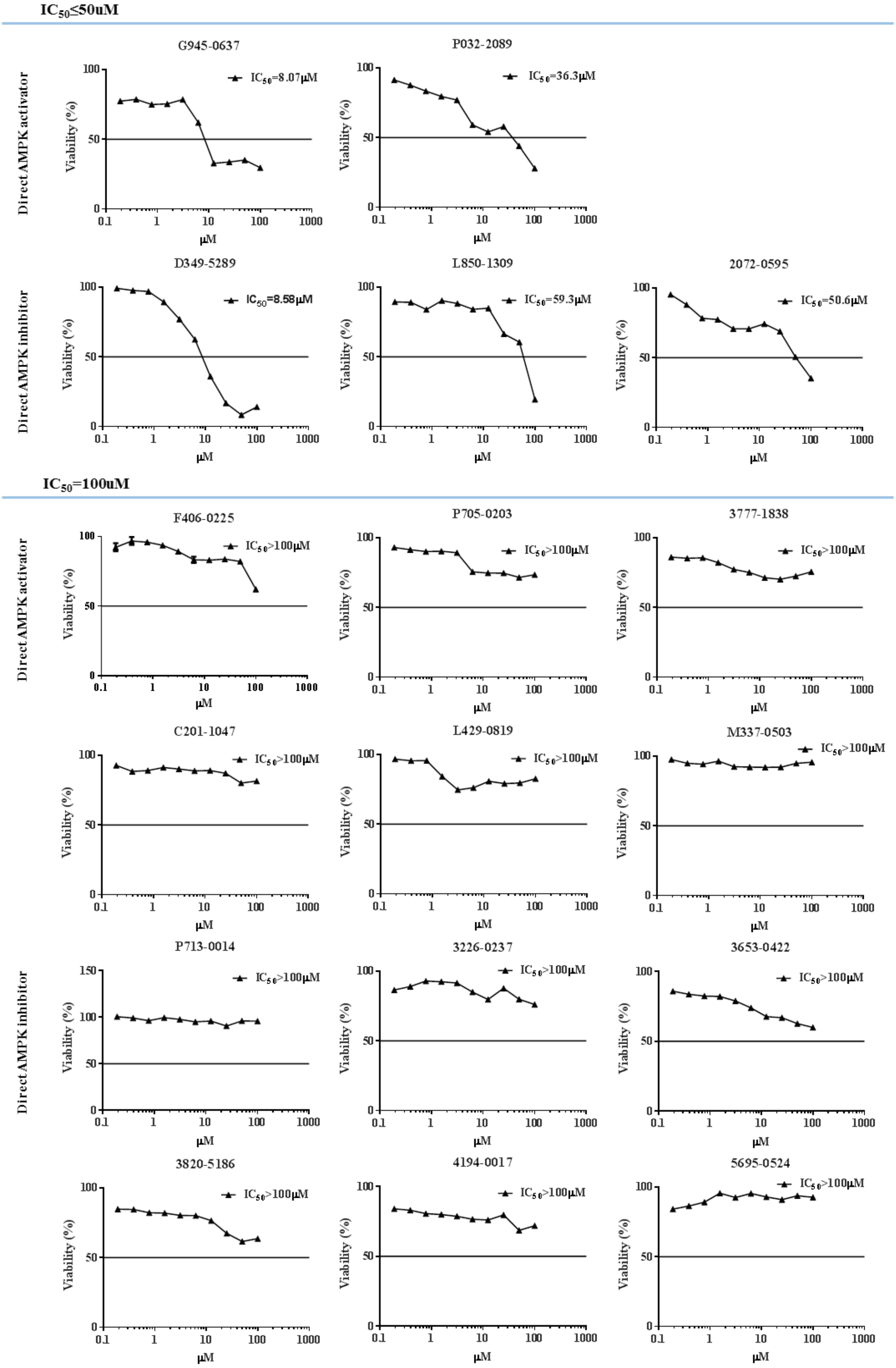

Figure S1. Analysis of HeLa cells viability after treatment with the examined compounds using IC_{50} cytotoxicity test. Compounds were classified as cytotoxic with IC_{50}≤50μM and non-cytotoxicity with IC_{50}=100μM, respectively.

Supplementary Figure 2

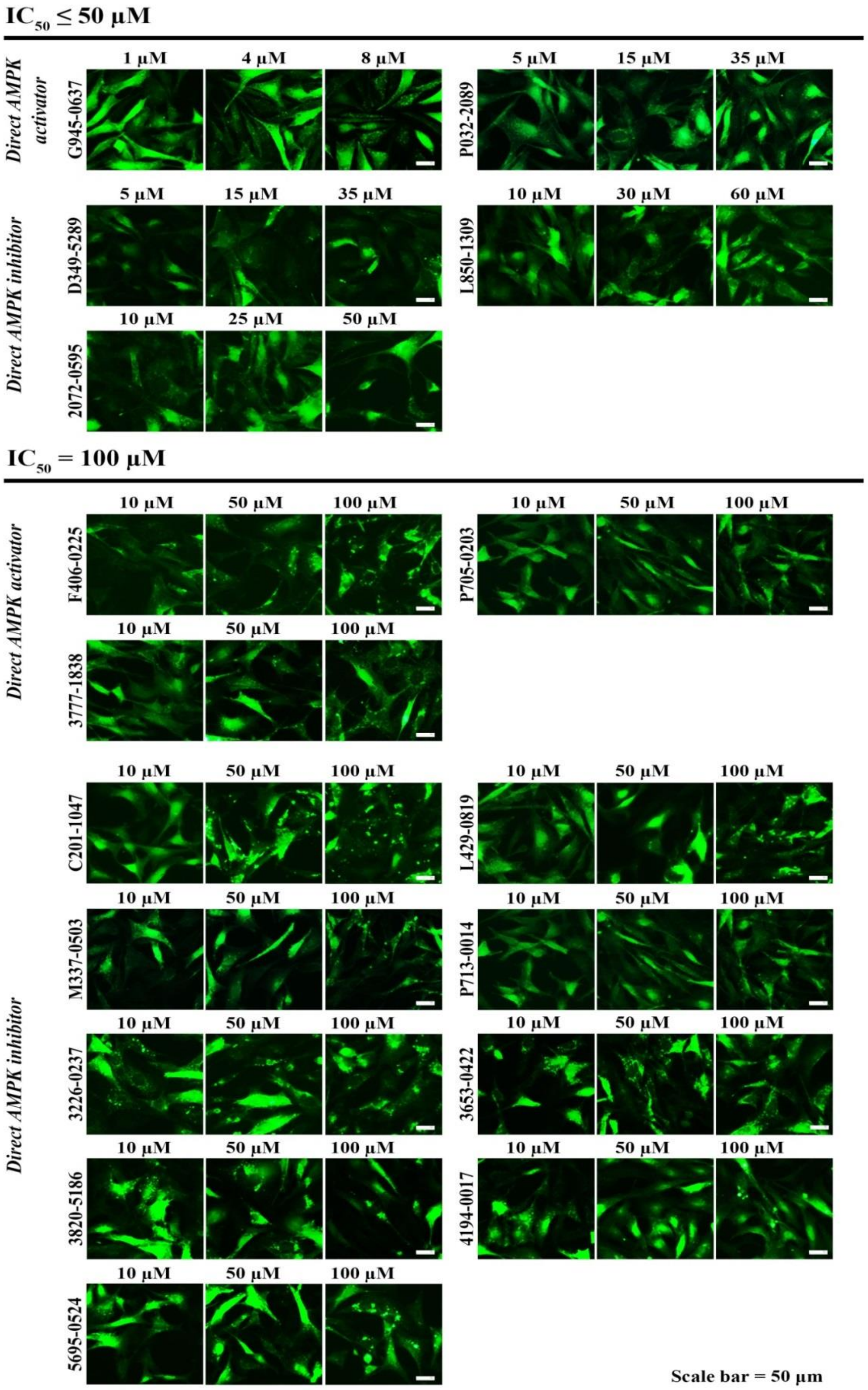

Figure S2. Immunofluorescence imaging analysis of autophagy activation in HeLa cells after treatment with the examined compounds. All of the compounds induce autophagosome formation (green puncta) in a dose-dependent manner.

Supplementary Figure 3

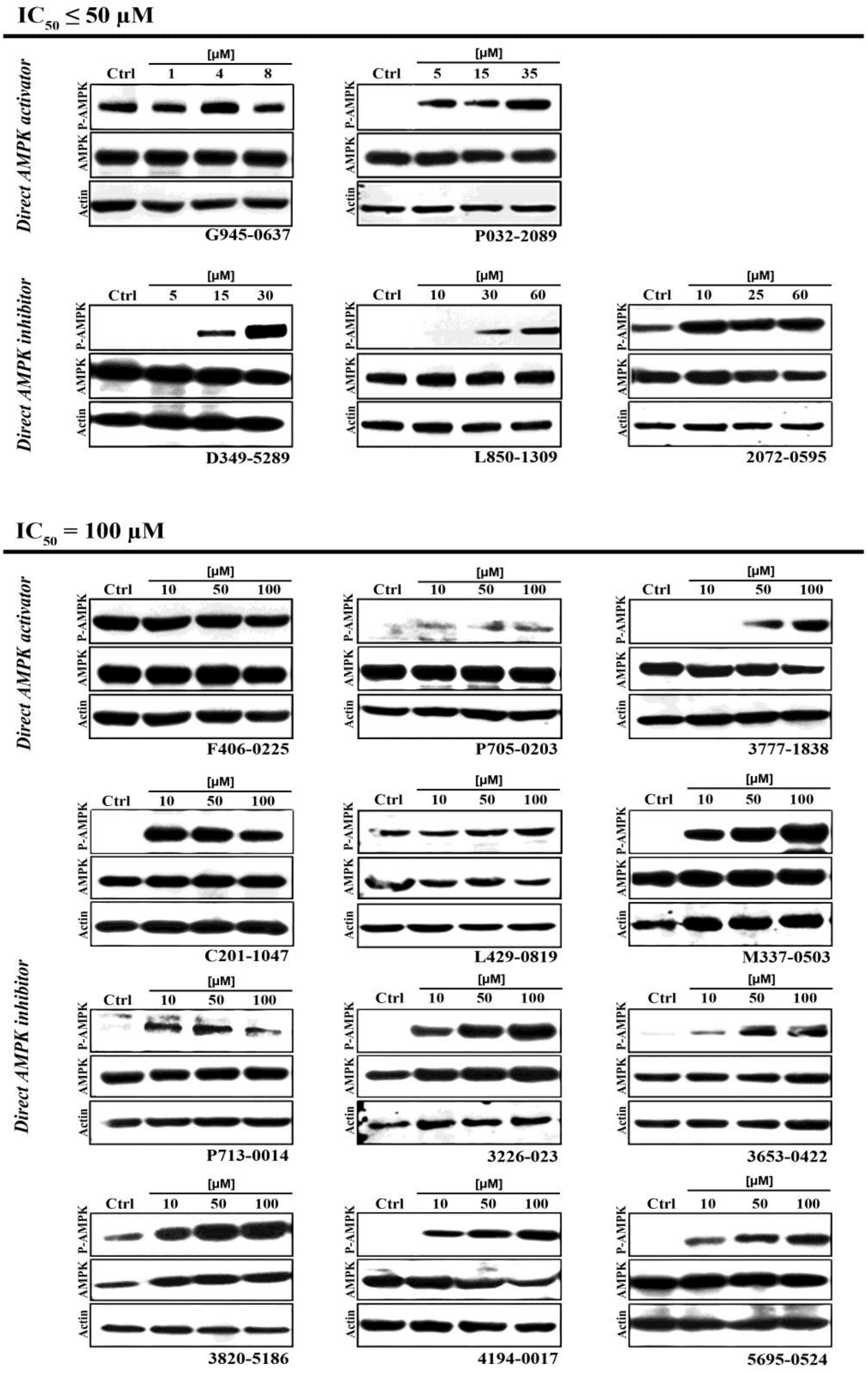

Figure S3. Western blot analysis of the phosphorylation profile of AMPK isolated from HeLa cells treated with the examined compounds. Most of the compounds induce the phosphorylation of AMPK in vivo in a dose-dependent manner.
